# Upper Limb Robotic Rehabilitation for Patients with Cervical Spinal Cord Injury: A Comprehensive Review

**DOI:** 10.3390/brainsci11121630

**Published:** 2021-12-10

**Authors:** Giovanni Morone, Alessandro de Sire, Alex Martino Cinnera, Matteo Paci, Luca Perrero, Marco Invernizzi, Lorenzo Lippi, Michela Agostini, Irene Aprile, Emanuela Casanova, Dario Marino, Giuseppe La Rosa, Federica Bressi, Silvia Sterzi, Daniele Giansanti, Alberto Battistini, Sandra Miccinilli, Serena Filoni, Monica Sicari, Salvatore Petrozzino, Claudio Marcello Solaro, Stefano Gargano, Paolo Benanti, Paolo Boldrini, Donatella Bonaiuti, Enrico Castelli, Francesco Draicchio, Vincenzo Falabella, Silvia Galeri, Francesca Gimigliano, Mauro Grigioni, Stefano Mazzoleni, Stefano Mazzon, Franco Molteni, Maurizio Petrarca, Alessandro Picelli, Marialuisa Gandolfi, Federico Posteraro, Michele Senatore, Giuseppe Turchetti, Sofia Straudi

**Affiliations:** 1 IRCCS Santa Lucia Foundation, 00179 Rome, Italy; a.martino@hsantalucia.it; 2 Physical and Rehabilitative Medicine, Department of Medical and Surgical Sciences, University of Catanzaro “Magna Graecia”, 88100 Catanzaro, Italy; 3 AUSL (Unique Sanitary Local Company), 50123 Florence, Italy; matteo.paci@applicazione.it; 4 Neurorehabilitation Unit, Azienda Ospedaliera SS. Antonio e Biagio e Cesare Arrigo, 15121 Alessandria, Italy; lperrero@ospedale.al.it; 5 Physical and Rehabilitative Medicine, Department of Health Sciences, University of Eastern Piedmont “A. Avogadro”, 10121 Novara, Italy; marco.invernizzi@med.uniupo.it (M.I.); lorenzolippi.mt@gmail.com (L.L.); 6 Translational Medicine, Dipartimento Attività Integrate Ricerca e Innovazione (DAIRI), Azienda Ospedaliera Nazionale SS. Antonio e Biagio e Cesare Arrigo, 15121 Alessandria, Italy; 7 Section of Rehabilitation, Department of Neuroscience, University General Hospital of Padova, 35128 Padua, Italy; michela.agostini@unipd.it; 8 IRCCS Fondazione Don Carlo Gnocchi, 50123 Florence, Italy; iaprile@dongnocchi.it; 9 IRCCS Istituto delle Scienze Neurologiche di Bologna, UOC Medicina Riabilitativa e Neuroriabilitazione, 40139 Bologna, Italy; e.casanova@ausl.bologna.it (E.C.); alberto.battistini@ausl.bologna.it (A.B.); 10 IRCCS Neurolysis Center “Bonino Pulejo”, 98124 Messina, Italy; dario.marino95@gmail.com; 11 C.S.R.—Consorzio Siciliano di Riabilitazione, 95123 Catania, Italy; laros.giu@gmail.com; 12 Campus Bio-Medico University Hospital, University of Rome, 00128 Rome, Italy; b.federica@unicampus.it (F.B.); s.sterzi@unicampus.it (S.S.); s.miccinilli@unicampus.it (S.M.); 13 National Center for Innovative Technologies in Public Health, Italian National Institute of Health, 00161 Rome, Italy; daniele.giansanti@iss.it (D.G.); mauro.grigioni@iss.it (M.G.); 14 Padre Pio Foundation and Rehabilitation Center, San Giovanni Rotondo 71013, Italy; serena.diba@gmail.com; 15 A.O.U. Città della Salute e della Scienza di Torino, 10126 Turin, Italy; monicasicari80@gmail.com (M.S.); salvatore.petrozzino@gmail.com (S.P.); 16 CRRF “Mons. Luigi Novarese”, 13040 Moncrivello, Italy; csolaro@libero.it; 17 Fondazione Don Carlo Gnocchi, 10143 Torino, Italy; stgargano@dongnocchi.it; 18 Department of Moral Theology, Pontifical Gregorian University, 00187 Rome, Italy; benanti@unigre.it; 19 Società Italiana di Medicina Fisica e Riabilitativa (SIMFER), 00198 Rome, Italy; paolobold@gmail.com (P.B.); dbonaiuti2@yahoo.it (D.B.); 20 Paediatric Neurorehabilitation Department, IRCCS Bambino Gesù Children’s Hospital, 00163 Rome, Italy; enrico.castelli@opbg.net; 21 Department of Occupational and Environmental Medicine, Epidemiology and Hygiene, INAIL, Monte Porzio Catone, 00185 Rome, Italy; f.draicchio@inail.it; 22 Italian Federation of Persons with Spinal Cord Injuries (Faip Onlus), 00195 Rome, Italy; falabella@fishonlus.it; 23 IRCCS Fondazione Don Carlo Gnocchi, 20148 Milan, Italy; sgaleri@dongnocchi.it; 24 Multidisciplinary Department of Medicine for Surgery and Orthodontics, University of Campania “Luigi Vanvitelli”, 80138 Naples, Italy; francescagimigliano@gmail.com; 25 Department of Electrical and Information Engineering, Politecnico di Bari, 70125 Bari, Italy; stefano.mazzoleni@poliba.it; 26 AULSS6 (Unique Sanitary Local Company) Euganea Padova, Rehabilitation Department, 35128 Padua, Italy; stefano.mazzon@gmail.com; 27 Villa Beretta Rehabilitation Center, Department of Rehabilitation Medicine, Valduce Hospital, 23845 Costa Masnaga, Italy; fmolteni@valduce.it; 28 Movement Analysis and Robotics Laboratory MARlab, IRCCS Bambino Gesù Children’s Hospital, 00163 Rome, Italy; maurizio.petrarca@opbg.it; 29 Department of Neurosciences, Biomedicine and Movement Sciences, University of Verona, 37129 Verona, Italy; alessandro.picelli@univr.it (A.P.); marialuisa.gandolfi@univr.it (M.G.); 30 Rehabilitation Department Versilia Hospital, Versilia Hospital AUSL Toscana Nord Ovest, 55049 Lido di Camaiore, Italy; federico.posteraro@uslnordovest.toscana.it; 31 AITO (Associazione Italiana Terapisti Occupazionali), 00136 Rome, Italy; presidente@aito.it; 32 Management Institute, Sant’Anna School of Advanced Studies, 56127 Pisa, Italy; giuseppe.turchetti@santannapisa.it; 33 Neuroscience and Rehabilitation Department, Ferrara University Hospital, 44121 Ferrara, Italy; sofia.straudi@gmail.com

**Keywords:** cervical spinal cord injury, arm function, exoskeleton, robot-assisted therapy, robotic therapy, rehabilitation

## Abstract

The upper extremities limitation represents one of the essential functional impairments in patients with cervical spinal cord injury. Electromechanics assisted devices and robots are increasingly used in neurorehabilitation to help functional improvement in patients with neurological diseases. This review aimed to systematically report the evidence-based, state-of-art on clinical applications and robotic-assisted arm training (RAT) in motor and functional recovery in subjects affected by cervical spinal cord injury. The present study has been carried out within the framework of the Italian Consensus Conference on “Rehabilitation assisted by robotic and electromechanical devices for persons with disability of neurological origin” (CICERONE). PubMed/MEDLINE, Cochrane Library, and Physiotherapy Evidence Database (PEDro) databases were systematically searched from inception to September 2021. The 10-item PEDro scale assessed the study quality for the RCT and the AMSTAR-2 for the systematic review. Two different authors rated the studies included in this review. If consensus was not achieved after discussion, a third reviewer was interrogated. The five-item Oxford CEBM scale was used to rate the level of evidence. A total of 11 studies were included. The selected studies were: two systematic reviews, two RCTs, one parallel-group controlled trial, one longitudinal intervention study and five case series. One RCT was scored as a high-quality study, while the systematic review was of low quality. RAT was reported as feasible and safe. Initial positive effects of RAT were found for arm function and quality of movement in addition to conventional therapy. The high clinical heterogeneity of treatment programs and the variety of robot devices could severely affect the generalizability of the study results. Therefore, future studies are warranted to standardize the type of intervention and evaluate the role of robotic-assisted training in subjects affected by cervical spinal cord injury.

## 1. Introduction

Spinal cord injury (SCI) represents one of the most disabling neurological conditions by complete or incomplete damage to the spinal cord with resulting detrimental consequences in motor, sensitive, and visceral controls [1,2,3,4].

The prevalence of SCIs widely varies among countries, ranging from 13.0 per million to 163.4 per million people [5]. Considering that most of the presentation involves young adults, both sanitary costs and lifetime assistance costs are highly burdensome, estimating a comprehensive cost of more than 1 million dollars per person [6]. SCIs might arise from mechanical damages (i.e., contusions, compressions or lacerations of the spinal cord) or non-traumatic events (e.g., degenerative cervical myelopathies, cancers, infections, intervertebral disc diseases, etc.) [6,7].

High-level spinal cord lesions could lead subjects to a high disability, considering the loss of arms and hands function related to detrimental consequences of functional impairment, reduced independence in activities of daily living (ADL), and a poor Health-Related Quality of Life (HRQoL) [1,2,7,8].

Rehabilitation might play a crucial role in the arm and hand functional recovery of patients affected by SCI, with a large variety of therapeutic options currently adopted. [7,9] It has been recently proposed that repetitive, task-specific, functional training could be considered effective in improving upper limb functions, even potentially interacting with the self-repair capacity of the spinal cord [10,11].

Among the new therapeutic options, robotic devices are well suited to produce intensive, task-oriented motor training that might enhance conventional rehabilitation facilitating the plasticity-related recovery by increasing sensory feedback and supporting the motor system [12].

These devices might perform arm or hand-assisted training, typically targeting either the shoulder and elbow, or the wrist and fingers. Robotic devices can be categorized as exoskeletons or end-effectors. Exoskeletons are devices that directly control the articulation of targeted joint(s), whereas robotic end-effectors contact users at the distal part of their limb [11,13,14]. Robotic devices are currently used in clinical practice to deliver an adequate intensity of training in terms of movement repetitions even in more severe subjects, which promotes functional recovery and may potentially facilitate adaptive plasticity [11,13].

In addition, robotic training provides the standardized rehabilitative training and monitors recovery of motor function in patients more objectively, thus reducing the subjective human influence [15]. Robotic rehabilitation aims to optimize learning strategies and to provide a patient-tailored rehabilitation plan [11]. Nowadays, more than 120 devices have been developed for upper limb rehabilitation of patients affected by neurologic disability [16].

To date, interest has been growing in the scientific literature, with several papers suggesting medical relevant features of robotic-assisted rehabilitation in functional recovery of patients affected by neurologic disability [14,17,18,19]. However, despite these promising findings, there is not agreement on the effectiveness of this novel approach in the current clinical practice of the rehabilitation field. Moreover, even the expensive technology could limit the spreading of this advanced treatment in clinical settings and the evidence of its effectiveness in patients affected by neurological diseases of rehabilitative interest, including SCI.

Therefore, this comprehensive review of systematic reviews and clinical studies summarizes the state-of-art on safety, clinical applications, and effectiveness of robotic rehabilitation in the integrated management of upper limb functional recovery in SCI patients.

## 2. Materials and Methods

The present study has been carried out within the framework of the Italian Consensus Conference on “Rehabilitation assisted by robotic and electromechanical devices for persons with disability of neurological origin” (CICERONE) [20].

### 2.1. Search Strategy

PubMed/MEDLINE, Cochrane Library, and Physiotherapy Evidence Database (PEDro) databases were systematically searched from inception to September 2021 for all the papers published following the SPIDER tool strategy [21], depicted by Table 1.

This comprehensive systematic review of systematic reviews and clinical studies has been performed in accordance with the Preferred Reporting Items for Systematic Reviews and Meta-analyses (PRISMA) statement. [22]

### 2.2. Selection Criteria

After the ‘duplicates’ removal, two reviewers (LP, LL) independently screened for inclusion title and abstract of all potentially relevant studies identified. In case of disagreement, a consensus was achieved by the decision of a third reviewer (AdS). Full-text studies were retrieved by the same two reviewers (LP, LL) and independently screened for inclusion. If consensus was not achieved by discussion between them, disagreements were solved by the decision of a third reviewer (AdS).

Randomized controlled trials were considered eligible if responding to the questions defined according to the following PICO model: (P) Participants: SCI patients in acute, subacute (≤3 months after injury), or chronic phase; (I) Intervention: Rehabilitation training with robotic-assisted devices for upper limb, with or without conventional therapy; (C) Comparator: Conventional rehabilitation; (O) Outcome measures: safety of robotic rehabilitation, the feasibility of robotic rehabilitation, upper limb strength, functioning, independence in ADL, and HRQoL.

We included systematic reviews, randomized controlled trials (RCTs), observational analytic studies, and case series. Exclusion criteria were: (1) papers involving animals; (2) language other than English; (3) case reports design; (4) participants with different neurologic disabilities from SCI; (5) robotic-assisted rehabilitation combined with other advanced technologies such as non-invasive brain stimulations (NIBS) or transcranial direct current stimulation (tDCS).

### 2.3. Data Extraction and Synthesis

All data were extracted from eligible full-text documents through Excel by two different authors. In case of disagreement, the consensus was achieved by the review of a third author.

The following data were extracted: (1) title; (2) authors; (3) publication year; (4) study design; (5) participants; (6) intervention characteristics; (7) outcomes; (8) main findings.

All studies included were synthesized, describing both study characteristics and data extracted. A meta-analysis was not performed given the high clinical heterogeneity in design, intervention, and outcomes assessed in the different studies.

### 2.4. Study Quality

The five-item Oxford CEBM scale was used to rate the level of evidence (OCEBM website). The study quality included was assessed by the 16-item assessment of multiple systematic reviews 2 (AMSTAR 2) scale [23] for systematic reviews, and the 10-item PEDro scale24 for the randomised clinical trials. Regarding the PEDro scale, the risk of bias was rated as poor (0–3), fair (4–5), good (6–8) and excellent (9–10) in line with the PEDro scale. [24] Two different authors rated the studies included in this systematic review. If consensus was not achieved after discussion, a third reviewer was interrogated.

## 3. Results

### 3.1. Evidence Synthesis

**Figure 1:**
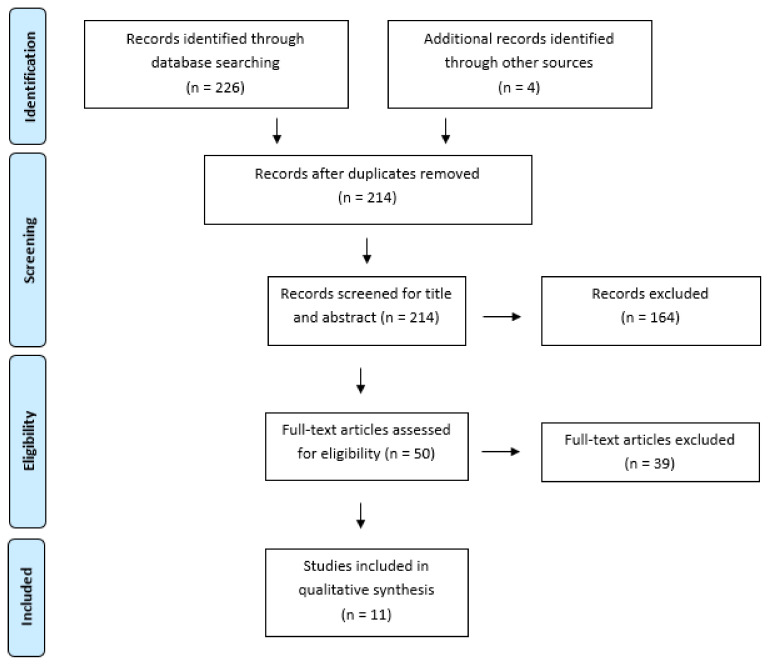
Prisma Flow chart.

Out of 226 studies identified from the databases, 214 were considered eligible for inclusion after duplicate removal and screened for title and abstract: 164 were excluded, and 50 full-text papers were screened. Subsequently, 39 articles were excluded because they did not respect eligibility criteria. As a result, 11 papers [25,26,27,28,29,30,31,32,33,34,35] were included in the qualitative synthesis (PRISMA flow diagram was depicted by Figure 1): five case series [25,26,27,28,29], one parallel-group controlled trial [30], two RCT [31,34], two systematic reviews [32,33] and one longitudinal intervention study [35].

The studies included in this systematic review were published from 2012 [25] to 2020 [35], covering several Nations from all over the world; more in detail, seven studies were from the Americas (two from Canada [25,32] and five from USA [26,27,29,30,33]), two from Europe (one from Netherlands [28] and one from UK [35]), and two from Asia (Republic of Korea [31,34]).

### 3.2. Evidence Level and Study Quality of the Included Studies

Due to the high clinical heterogeneity of the included studies; thus, the results are described qualitatively. Based on the Oxford Centre for Evidence-Based (OCEBM) 2011 Levels of Evidence, [34] we included two systematic reviews [32,33] (Level 1), 2 RCT [31,34] (Level 2), one parallel-group controlled trial [30] (Level 3), one longitudinal intervention trial [35] (Level 3), and five case series (Level 4) [25,26,27,28,29].

The study cohort sample sizes were highly heterogeneous in the research studies, ranging from five (case series) [28] to 34 (RCT) [31] for clinical trials; nevertheless, the systematic reviews included larger samples (73 study participants by Singh et al. [32] and 88 by Yozbatiran et al. [33]). All the studies assessed patients of both genders, with ages ranging from 17 [26] to 76 years. [29] The study by Fitle et al. [27] did not report age.

Concerning the study quality of the clinical studies, we reported one good-quality [31], one fair-quality [34], according to the PEDro scale [24]. The two systematic reviews showed a low quality [31] and a critically low quality [33] according to AMSTAR 2 scale [23].

### 3.3. Clinical Characteristics of Study Participants

Six studies included SCI patients in the chronic phase [26,27,28,29,30,34], two in the subacute phase [25,35], and three papers [31,32,33] included both chronic and subacute SCI patients. Complete (American Spinal Injury Association Impairment Scale—AIS—A and B) and incomplete lesions (AIS C and D) were assessed by seven studies [25,26,28,31,32,33,34] while four studies [27,29,30,35] selected only incomplete lesions (AIS C and D). All the clinical trials included clarified SCI levels, ranging between C2 [29] and C8 [31] (further details are depicted in Table 2).

### 3.4. Robotic Rehabilitation Characteristics

Robotic devices assessed in the studies included resulted to be extremely heterogeneous. Armeo Spring [25,32,33], InMotion 3.0 Wrist robot [26,32], Haptic Master [28,32], MAHI Exo-II [27,29,30,33], Armeo Power [31,34], RiceWrist-S [33], Reo Go, [32,33] Haptic Master, [32,33] Reaching Robot, [32,33] Amodeo, [34] SEM Glove. [35]

The joints involved with robotic training were: shoulder, [25,34] elbow, [25,27,29,30,31,34] wrist [25,27,29,30,31,34], and fingers [31,34,35]. Even robotic-assisted rehabilitation programs were heterogeneous, varying from 429 [31], to 12 weeks [35], with the duration of the interventions ranging from a total of 30 min [31] to 4 h per day [35]. Training sessions ranged from 130 to 5 per week [25]. On the other hand, supervision was not clarified by two study [26,34], one did not perform a supervision [35], whereas all the other research studies included assessed supervised exercise programs [25,27,28,29,30,31]. Robot-assisted training was assessed as an add-on conventional therapy in four studies, [25,28,31,34], whereas five studies considered stand-alone robotic training [26,27,29,30,35]. Only two studies compared occupational therapy with occupational therapy combined with robotic training [31,34]. Moreover, Zariffa et al. [25] compared the efficacy of the unilateral treatment with the contralateral upper limb. Lastly, both systematic reviews included studies with robotic training combined or not combined with conventional therapy (further details on robotic rehabilitation in the included studies are depicted by Table 2) [32,33].

### 3.5. Main Findings of the Included Studies

All the case series [25,26,27,28,29] included in the present systematic review assessed the feasibility of robotic rehabilitation in SCI patients. Zariffa et al. [25] assessed both compliance and therapist timing, reporting that more rehabilitation exercises were performed with progressively less hands-on involvement by the therapist. Tolerance has been assessed by Francisco et al. [29], reporting no significant increase of self-reported pain and discomfort level during the therapy sessions. Accordingly, Cortes et al. [26] reported a high safety profile and tolerance without increasing pain and spasticity, and Vanmulken et al. [28] showed a discrete tolerance (Usefulness, Satisfaction and Ease-of-use questionnaire mean score of 66.1 ± 14.7%). Lastly, it should be highlighted that all papers included in this systematic review [25,26,27,28,29,30,31,32,33] did not report any major adverse event during robot-assisted training in SCI patients.

A main rehabilitative measure as muscle strength was assessed by all the research articles, albeit with a wide heterogeneity in terms of outcomes, including Medical Research Council grade, [29] Manual Muscle Test (MMT), [31] grip [25,29] and pinch [29] strength, and upper extremity motor score (UEMS) [25]. Two studies [25,26] reported no significant changes in terms of muscle strength; on the contrary, Francisco et al. [29] showed a significant improvement of muscle strength (UEMS: 31.5 ± 2.3 vs. 34.0 ± 2.3; *p* = 0.04; grip strength: 9.7 ± 3.8 vs. 12 ± 4.3; *p* = 0.02; pinch strength 4.5 ± 1.1 vs. 5.7 ± 1.2; *p* = 0.01), even maintained at follow-up evaluation (UEMS: 35.5 ± 2.0; *p* = 0.02; grip strength: 12.7 ± 4.0; *p* = 0.05; pinch strength 5.6 ± 1.2; *p* = 0.02).

Kim et al., [31] reported a significant improvement in terms of UEMS in the robotic training group compared to the control group (*p* = 0.03). However, no significant changes in MRC scale were shown. In particular, elbow flexors (C5) (*p* = 0.21), wrist extensors (C6) (*p* = 0.08), elbow extensors (C7) (*p* = 0.16), finger flexors (*p* = 0.66), and 5th finger abductors (T1) (*p* = 0.59).

In line with previous findings, both systematic reviews [32,33] affirmed that evidence supporting robot-assisted training effectiveness in muscle strength improvement in SCI patients is still controversial.

Concerning functioning, several outcome measures were assessed by the included papers, including graded and redefined assessment of strength, sensibility, and prehension (GRASSP) [25,30], Action Research Arm Test (ARAT) [25,27,29,30], Jebsen-Taylor Hand Function Test (JTHFT) [27,29], SCIM II [29], and SCIM III [31]. Zariffa et al. [25] showed a significant improvement of GRASSP score only in the subgroup with partial hand function at baseline (6.0 ± 1.6 vs. 1.9 ± 0.9; *p* = 0.04). Considering the whole sample, no significant results (*p* > 0.05) were underlined in both GRASSP scores. On the contrary, Frullo et al. [30] reported significant results in GRASSP strength (*p* = 0.031) and GRASSP sensation (0.002), although these results have not been corrected for multiple comparisons. No significant effects were shown in the ARAT score (*p* = 0.128). However, Francisco et al. [29], in their case series, reported a significant increase in terms of ARAT (30.7 ± 3.8 vs. 34.3 ± 4.0; *p* = 0.02) and JTHFT (0.14 ± 0.04 vs. 0.21 ± 0.07; *p* = 0.04), whereas SCIM II did not significantly improve (62.1 ± 9.7 vs. 62.6 ± 9.7; *p* = 0.18).

On the other hand, the RCT performed by Kim et al. reported significant differences between groups in terms of total SCIM-III score (7 [2 to 11] vs. 0 [−4 to 4]; *p* < 0.01). However, only the mobility (room and toilet) item significantly varied between groups (1 [0 to 3] vs. 0 [−1 to 1]; *p* = 0.02) in contrast with the other items not showing significant differences [31].

Both systematic reviews [32,33] reported that robot-assisted rehabilitation might be considered promising training to improve muscle function in SCI.

Lastly, the case series performed by Cortes et al. [26] evaluated kinematics and corticospinal excitability after robotic rehabilitation in SCI patients. The authors reported a significant improvement of kinematic (1.17 ± 0.11 radians vs. 1.03 ± 0.08 radians; *p* = 0.03) and smoothness of movement (0.26 ± 0.03 vs. 0.31 ± 0.02; *p* = 0.03) in SCI patients. However, the corticospinal excitability did not show significant changes (amplitude: 32 ± 0.5 mV vs. 27 ± 0.06 mV; *p* = 0.35; latency: 17.4 ± 0.7 ms vs. 16.9 ± 0.74 ms; *p* = 0.28). Similarly, Fitle et al. [27] showed a significant improvement between pre- and post-intervention in the non-segmental kinematic measure (normalized speed) of the less affected arm (*p* = 0.01). In addition, segmental kinematic measures improved significantly in the more affected arm (*p* = 0.03).

Lastly, the study by Frullo et al. [30] reported a significant improvement of normalized speed (*p* < 0.001), mean arrest period ratio (*p* = 0.001), and spectral arc length (*p* = 0.001) only in the assist-as-need group.

## 4. Discussion

Advancement in technology has been widely spreading in the rehabilitation field during the past two decades, and SCI patients might benefit from robotic rehabilitation. However, despite this approach being commonly adopted in the clinical practice, this systematic review showed that only a few studies assessed the effectiveness of robotic-assisted training for recovering upper limb muscle strength and function in patients with SCI.

Taken together, our findings suggested that robotic devices for upper limbs might be considered safe, tolerable, and feasible in the complex rehabilitative management of SCI patients. However, to date, safety, tolerance, and feasibility of robot-assisted training have been primarily investigated in patients with other neurological diseases (i.e., stroke and multiple sclerosis) [36,37,38] and these outcomes should be deeply assessed in SCI patients, starting from the findings reported by the present systematic review.

We highlighted that robotic rehabilitation mainly was assessed in patients suffering from incomplete SCI, both with sub-acute [25,31,32,33,34] and chronic lesions. [26,27,28,29,30,31,32,33,35] Among the included studies, Zariffa et al. [25] suggested that SCI patients with more represented residual function could beneficiate from robot-assisted therapy more than other subjects.

Indeed, motor and sensory feedback stimuli are key components of task-oriented robotic training and might be more effective in patients with incomplete SCI than complete SCI [11,12,13,14]. Moreover, plasticity process can be elicited indirectly by sensory and motor afferent stimuli and directly through neuromodulation via non-invasive brain stimulation. More in detail, Yozbatiran et al. [39] suggested that modulating excitatory input of the corticospinal tracts on spinal circuits induced by tDCS combined with robot-assisted training could improve arm and hand functions in persons with incomplete SCI. This intriguing study has not been included in our systematic review, considering that the combination of robotic-assisted rehabilitation with other advanced technologies (i.e., NIBS and tDCS) was an exclusion criterion due to the limitation that they might affect the efficacy of robot-assisted training. However, we are aware that this combination should be deeply investigated in future studies on SCI patients.

Robotic training should be considered as an “add on” to conventional therapy in sub-acute SCI patients (≤3 months after injury); four studies included in this systematic review assessed the role of robotic-assisted rehabilitation combined with conventional physical therapy [25,28] and occupational therapy [25,31], probably due to complex scenario underpinning SCI management. In contrast, robotic treatment has been proposed as a stand-alone therapy in three case series out of four involving chronic SCI patients [26,27,29].

The present comprehensive systematic review showed a lack of evidence on differences between proximal (shoulder elbow) and distal (hand) training according to the robot design. More in detail, rehabilitation robots could be classified into two groups: end-effector based robots, which provide training capability encapsulating a large portion of the functional workspace, and exoskeletons, designed to resemble human anatomy with a structure enabling individual actuation of joints [40]. Therefore, we would like to highlight that future studies should involve enhanced control modes to allow additional treatment options in SCI patients; indeed, taking into account the different actions that the upper limb might exert (i.e., reaching and grasping), robotic devices might have a more targeted function with a more specific mechanical design in order to perform an adequate patient-tailored rehabilitation in subjects after SCI.

Concerning the type of intervention proposed, very high variability was recorded in terms of robot devices, the number of sessions per day, session duration, frequency, and joint involvement. This intrinsic limitation, probably related to the first phase of adopting new technology, severely affects the generalizability of these findings. In addition, it should be noted that the type of treatment intervention should be based on the SCI level, considering the clinical heterogeneity of functional disability occurring in cervical SCI. Future studies should focus on larger samples involving cervical SCI patients divided into subgroups to provide a patient-tailored robotic rehabilitative treatment.

In the literature, we found two similar systematic reviews investigating the role of robotic rehabilitation in SCI patients, albeit their quality was classified as low [32] and very low, [33] according to AMSTAR 2 scale [23]. Indeed, both Smith et al. [32] and Yozbatiran et al. [33] summarized the available literature on the robot-assisted training in upper limb rehabilitation of SCI patients, including even case reports and studies on the combination of robotic rehabilitation with other advanced technologies, severely affecting the homogeneity of data assessed and heavily influencing their results.

Nevertheless, by the present systematic review, the RCT performed by Kim et al. [31] was investigated first. This good-quality paper reported a significant improvement in terms of UEMS in the robotic training group compared to the control group (1 [0 to 3] vs. 0 [−1 to 1]; *p* = 0.03) in SCI patients; on the other hand, no significant changes in MRC scale were shown (*p* > 0.05). The authors suggested that significant improvement in muscle strength might have potential benefits in terms of short-distance mobility and electrical wheelchair manipulation. In line with these findings, significant improvements in SCIM-III scores (7 [2 to 11] vs. 0 [−4 to 4]; *p* < 0.01) in the robot-assisted rehabilitation group might have positive effects in terms of independence in the ADL [31].

Considering these findings, the present study might be viewed as the first systematic review performed by a large consensus panel of experts, including research studies specifically assessing the effects of robot-assisted training of the upper limb in patients with SCI. We showed that the current available literature on this topic might be defined as low-quality evidence. The lack of evidence might be partly due to the rapid evolution of advanced technologies with high costs that might not allow a standardization and reproducibility of single large-scale rehabilitation intervention.

The studies included in this systematic review had several limitations, as the small sample sizes, [25,26,31] the lack of a control group [25,26,27,28,29], the monocentric design, [26,27,30,31] and the lack of long-term follow up evaluations [25,26,27,28,31]; as well as the wide variability in robotic devices, training protocols, and outcome measures adopted in the studies.

## 5. Conclusions

Taken together, the present comprehensive systematic review summarized the state-of-the-art robotic-assisted rehabilitation treatments available for patients suffering from cervical SCI. Nowadays, robotic-assisted training is still experimental, but recent studies provided preliminary evidence showing intriguing positive effects on functional outcomes in SCI patients. We are aware that the high clinical heterogeneity of treatment programs and the variety of robot devices could severely affect the generalizability of the study results; therefore, future studies are warranted to standardize the type of intervention and evaluate the role of a robot-assisted training in the complex rehabilitation management of patients with SCI.

## Figures and Tables

**Table 1:** Spider tool search strategy.

S	PI	D	E	R
*Sample*	*Phenomenon of* *Interest*	*Design*	*Evaluation*	*Research Type*
Spinal Cord Injury	Robotic rehabilitation for upper limb motor recovery	Research article	Functional and/or rehabilitative outcomes	Qualitative
(Spinal Cord Injury[Title/Abstract]) OR Spinal Cord Injuries[MeSH Terms]	((((robot[Title/Abstract]) OR exoskeleton[Title/Abstract]) OR end-effector[Title/Abstract]) OR robotics[MeSH Terms]) OR Exoskeleton Device[MeSH Terms] AND (((((((upper limb[Title/Abstract]) OR upper extremity[Title/Abstract]) OR hand[Title/Abstract]) OR arm[Title/Abstract]) OR upper extremity[MeSH Terms]))		(((function[Title/Abstract]) OR rehabilitation [Title/Abstract]) OR recovery [Title/Abstract])	

**Table 2:** Main characteristics of the studies included in this systematic review.

Article	Nation	Design	Aim	Number Participants (Drop-Outs)	Gender and Age	SCI Stage	SCI According to AIS	SCI Level	Methodological Quality	CBIM
Zariffa 2012 [25]	Canada	Case Series	To assess the feasibility and efficacy of upper limb robotic rehabilitation device in subacute cervical SCI	15 (3)	14 M, 1 F 19–75 years	Subacute	AIS A (*n* = 2) AIS B (*n* = 4) AIS C (*n* = 1) AIS D (*n* = 5)	C4–C6	n/a	4
Cortes 2013 [26]	USA	Case Series	To assess feasibility, safety, and effectiveness of robotic-assisted training in chronic SCI	10 (0)	8 M, 2 F 17–70 years	Chronic	AIS A (*n* = 3) AIS B (*n* = 4) AIS C (*n* = 1) AIS D (*n* = 2)	C4–C6	n/a	4
Fitle 2015 [27]	USA	Case series	To assess feasibility and effectiveness of a robotic exoskeleton designed to train elbow, forearm and wrist movements	10 (2)	8 M, 2 F, age: NR	Chronic	AIS C–D (*n* = 2)	C2–C6	n/a	4
Vanmulken 2015 [28]	Netherlands	Case Series	To assess feasibility and effectiveness (arm-hand function and performance) of haptic robot technology	5 (2)	4 M, 1 F 25–70 years	Chronic	AIS A (*n* = 1) AIS B (*n* = 2)	C3–C7	n/a	4
Francisco 2017 [29]	USA	Case Series	To assess feasibility, tolerability, and effectiveness of robotic-assisted arm training	10 (2)	8 M, 2 F, 19–76 years	Chronic	AIS C (*n* = 4) AIS D (*n* = 4)	C2–C7	n/a	4
Frullo 2017 [30]	USA	Parallel group controlled trial	To assess feasibility of subject-adaptive robotic-assisted therapy: AAN vs. ST training modality	17 (3)	12 M and 2 F, 3 NR 53.5 years	Chronic	AIS C–D (*n* = 17)	C3–C8	n/a	4
Kim 2019 [31]	Republic of Korea	RCT	To assess the clinical efficacy of upper limb robotic therapy in people with tetraplegia	34 (4) RT: 17 (2) CT: 17 (2)	28 M, 6 F, RT: 56.7 ± 13.6 years CT: 47.1 ± 14.9 years	Subacute/Chronic	AIS A (*n* = 8) AIS B (*n* = 6) AIS C (*n* = 4) AIS D (*n* = 16)	C2–C8	8/10	2
Singh 2018 [32]	Canada	Systematic review	To summarize feasibility and outcomes of robotic-assisted upper extremity training for patients with cervical SCI	73 (11)	46 M, 8 F, 7 NR 17–75 years	Subacute/Chronic	AIS A-B (*n* = 16) AIS C-D (*n* = 46)	C2–C8	Critically low quality	3
Yozbatiran 2019 [33]	USA	Systematic review	To summarize the current evidence of robot-assisted rehabilitation in patients with tetraplegia	88 (13)	69 M, 13 F, 6 NR 17–76 years	Subacute/Chronic	AIS A–B (*n* = 14) AIS C–D (*n* = 58) 3 NR	C2–C7	Low quality	3
Jung 2019 [34]	Republic of Korea	RCT	To assess the effects of combined upper limb robotic therapy (RT) as compared to conventional occupational therapy (OT) in SCI patients	38 (8) RT: 22 (5) CT: 16 (3)	24 M, 6 F RT: 47.23 ± 14 CT: 53 ± 13.5	Subacute	AIS A (*n* = 3) AIS B (*n* = 4) AIS C (*n* = 7) AIS D (*n* = 16)	C2–C7	4/10	3
Osuagwu 2020 [35]	UK	Interventional longitudinal clinical trial design	To investigate the therapeutic effect of a self-administered home-based hand rehabilitation programme for people with cervical SCI using the soft extra muscle (SEM) Glove	15 (0)	11 M, 4 F 50.3 (33–60)	Chronic	AIS C (*n* = 3) AIS D (*n* = 11) Untested (*n* = 1)	C2–C5	n/a	4
Zariffa 2012 [25]	Canada	Case Series	To assess the feasibility and efficacy of upper limb robotic rehabilitation device in subacute cervical SCI	15 (3)	14 M, 1 F 19–75 years	Subacute	AIS A (*n* = 2) AIS B (*n* = 4) AIS C (*n* = 1) AIS D (*n* = 5)	C4–C6	n/a	4
Cortes 2013 [26]	USA	Case Series	To assess feasibility, safety, and effectiveness of robotic-assisted training in chronic SCI	10 (0)	8 M, 2 F 17–70 years	Chronic	AIS A (*n* = 3) AIS B (*n* = 4) AIS C (*n* = 1) AIS D (*n* = 2)	C4–C6	n/a	4
Fitle 2015 [27]	USA	Case series	To assess feasibility and effectiveness of a robotic exoskeleton designed to train elbow, forearm and wrist movements	10 (2)	8 M, 2 F, age: NR	Chronic	AIS C-D (*n* = 2)	C2–C6	n/a	4
Vanmulken 2015 [28]	Netherlands	Case Series	To assess feasibility and effectiveness (arm-hand function and performance) of haptic robot technology	5 (2)	4 M, 1 F 25–70 years	Chronic	AIS A (*n* = 1) AIS B (*n* = 2)	C3–C7	n/a	4
Francisco 2017 [29]	USA	Case Series	To assess feasibility, tolerability, and effectiveness of robotic-assisted arm training	10 (2)	8 M, 2 F, 19–76 years	Chronic	AIS C (*n* = 4) AIS D (*n* = 4)	C2–C7	n/a	4
Frullo 2017 [30]	USA	Parallel group controlled trial	To assess feasibility of subject-adaptive robotic-assisted therapy: AAN vs. ST training modality	17 (3)	12 M and 2 F, 3 NR 53.5 years	Chronic	AIS C–D (*n* = 17)	C3–C8	n/a	4
Kim 2019 [31]	Republic of Korea	RCT	To assess the clinical efficacy of upper limb robotic therapy in people with tetraplegia	34 (4) RT: 17 (2) CT: 17 (2)	28 M, 6 F, RT: 56.7 ± 13.6 years CT: 47.1 ± 14.9 years	Subacute/Chronic	AIS A (*n* = 8) AIS B (*n* = 6) AIS C (*n* = 4) AIS D (*n* = 16)	C2–C8	8/10	2
Singh 2018 [32]	Canada	Systematic review	To summarize feasibility and outcomes of robotic-assisted upper extremity training for patients with cervical SCI	73 (11)	46 M, 8 F, 7 NR 17–75 years	Subacute/Chronic	AIS A-B (*n* = 16) AIS C–D (*n* = 46)	C2–C8	Critically low quality	3
Yozbatiran 2019 [33]	USA	Systematic review	To summarize the current evidence of robot-assisted rehabilitation in patients with tetraplegia	88 (13)	69 M, 13 F, 6 NR 17–76 years	Subacute/Chronic	AIS A–B(*n* = 14) AIS C–D (*n* = 58) 3 NR	C2–C7	Low quality	3
Jung 2019 [34]	Republic of Korea	RCT	To assess the effects of combined upper limb robotic therapy (RT) as compared to conventional occupational therapy (OT) in SCI patients	38 (8) RT: 22 (5) CT: 16 (3)	24 M, 6 F RT: 47.23 ± 14 CT: 53 ± 13.5	Subacute	AIS A (*n* = 3) AIS B (*n* = 4) AIS C (*n* = 7) AIS D (*n* = 16)	C2–C7	4/10	3
Osuagwu 2020 [35]	UK	Interventional longitudinal clinical trial design	To investigate the therapeutic effect of a self-administered home-based hand rehabilitation programme for people with cervical SCI using the soft extra muscle (SEM) Glove	15 (0)	11 M, 4 F 50.3 (33–60)	Chronic	AIS C (*n* = 3) AIS D (*n* = 11) Untested (*n* = 1)	C2–C5	n/a	4

Abbreviations: AAN: assist-as-needed; AIS: American Spinal Injury Association Impairment Scale; CT: conventional therapy; F: Female; M: Male; NR: not reported; RCT: Randomized Controlled Trial; RT: robotic training; SCI: Spinal Cord Injury; ST: subject-triggered; USA: United States of America; CBIM.

## Data Availability

The dataset is available on request.
